# Eating Behavior, Physical Activity and Exercise Training: A Randomized Controlled Trial in Young Healthy Adults

**DOI:** 10.3390/nu12123685

**Published:** 2020-11-29

**Authors:** Wendy D. Martinez-Avila, Guillermo Sanchez-Delgado, Francisco M. Acosta, Lucas Jurado-Fasoli, Pauline Oustric, Idoia Labayen, John E. Blundell, Jonatan R. Ruiz

**Affiliations:** 1 PROmoting FITness and Health through Physical Activity Research Group (PROFITH), Sport and Health University Research Institute (iMUDS), University of Granada, 18007 Granada, Spain; gsanchezdelgado@ugr.es (G.S.-D.); acostaf@ugr.es (F.M.A.); juradofasoli@gmail.com (L.J.-F.); ruizj@ugr.es (J.R.R.); 2 Department of Physical Education and Sport, Faculty of Sport Sciences, University of Granada, 18011 Granada, Spain; 3 Pennington Biomedical Research Center, Baton Rouge, LA 70808, USA; 4 EFFECTS-262 Research Group, Department of Medical Physiology, School of Medicine, University of Granada, 18071 Granada, Spain; 5 Appetite Control and Energy Balance Group, School of Psychology, Faculty of Medicine and Health, University of Leeds, Leeds LS2 9JT, UK; pspjo@leeds.ac.uk (P.O.); j.e.blundell@leeds.ac.uk (J.E.B.); 6 Institute for Innovation & Sustainable Development in Food Chain (IS-FOOD), IDISNA, Navarra’s Health Research Institute (IdiSNA), Public University of Navarra, 31006 Pamplona, Spain; idoia.labayen@unavarra.es

**Keywords:** nutrition, appetite, binge eating, accelerometry, energy intake

## Abstract

Regular physical activity (PA) is an important part of the treatment of several medical conditions, including overweight and obesity, in which there may be a weakened appetite control. Eating behaviour traits influence weight control and may be different in active and sedentary subjects. This paper reports the relationships between the time spent in sedentary behaviour and physical activity (PA) of different intensity, and eating behaviour traits in young, healthy adults. Additionally, it reports the results of a six-month-long, randomized, controlled trial to examine the effect of an exercise intervention on eating behaviour traits. A total of 139 young (22.06 ± 2.26 years) healthy adults (68.35% women) with a Body Mass Index (BMI) of 24.95 ± 4.57 kg/m^2^ were enrolled. Baseline assessments of habitual PA were made using wrist-worn triaxial accelerometers; eating behaviour traits were examined via the self-reported questionnaires: Binge Eating, Three-Factor Eating Questionnaire-R18 and Control of Eating Questionnaire. The subjects were then randomly assigned to one of three groups: control (usual lifestyle), moderate-intensity exercise (aerobic and resistance training 3¨C4 days/week at a heart rate equivalent to 60% of the heart rate reserve (HRres) for the aerobic component, and at 50% of the 1 repetition maximum (RM) for the resistance component), or vigorous-intensity exercise (the same training but at 80% HRres for half of the aerobic training, and 70% RM for the resistance training). At baseline, sedentary behaviour was inversely associated with binge eating (*r =* −0.181, *p* < 0.05) and with uncontrolled eating (*r =* −0.286, *p* = 0.001). Moderate PA (MPA) was inversely associated with craving control (*r =* −0.188, *p* < 0.05). Moderate-to-vigorous PA (MVPA) was directly associated with binge eating (*r =* 0.302, *p* < 0.001) and uncontrolled eating (*r =* 0.346, *p* < 0.001), and inversely associated with craving control (*r =* −0.170, *p* < 0.015). Overall, PA was directly associated with binge eating (*r =* 0.275, *p* = 0.001), uncontrolled eating (*r =* 0.321, *p* < 0.001) and emotional eating (*r =* 0.204, *p* < 0.05). Additionally, only emotional eating was modified by the intervention, increasing in the vigorous-intensity exercise group (*p* < 0.05). In summary, we observed that time spent in sedentary behaviour/PA of different intensity is associated with eating behaviour traits, especially binge eating in young adults. In contrast, the six-month exercise intervention did not lead to appreciable changes in eating behaviour traits.

## 1. Introduction

Obesity is an important risk factor for the development of diabetes, cardiovascular disease and other non-communicable conditions. An increased energy intake, a sedentary lifestyle, and a lack of physical activity (PA) are the major causes of obesity [1]. Regular PA can contribute to improvements in energy and macronutrient balance [1], and is recommended for weight loss. However, PA increases energy expenditure, which can act as a driver of energy intake thereby inducing compensatory responses in energy intake and appetite regulation, commonly resulting in very little weight being lost.

Appetite influences the selection of food items, meal size and the frequency of eating [2]. However, eating behaviour is also influenced by internalised multidimensional traits that include behavioural, cognitive and affective components [3]. Since eating occurs in a variety of complex situations with varying social and cultural influences, energy intake can be notably altered by an individual’s psychological state and cognitive factors [4]. Restraint and disinhibition are two of the main cognitive characteristics that exert an effect on appetite regulation and food intake [5]. Dietary restraint refers to behaviours adopted to avoid weight gain (e.g., avoiding fattening foods, eating smaller portions), while disinhibition is the tendency towards overeating and eating opportunistically [6]. Further, food cravings and the urge to eat a certain type of food are components of the hedonistic control of appetite [7] which is activated by the thought of the sensorial pleasure of eating palatable food [5]. These eating behaviour traits can be measured through psychometric tools such as the Binge Eating Scale (BES) [8] for binge behaviour, the Three-Factor Eating Questionnaire (TFEQ) [9,10] for cognitive restraint, emotional eating and uncontrolled eating, and the Control of Eating Questionnaire (CoEQ) [11] for food cravings and mood.

Regular PA influences eating behaviour by enhancing the sensitivity of the physiological satiety signalling system, adjusting macronutrient food choices and requirements and changing the hedonic response to food stimuli [12,13]. However, low levels of PA do not drag down food consumption to match the associated low energy expenditure [14]. For instance, individuals who undertake only low-level PA are reported to have a weaker satiety response to food intake [13]. Drenowatz et al. indicate that specific types of exercise influence the frequency and intensity of food cravings [7]. However, it remains unclear how PA could be associated and influence eating behaviour traits in young adults.

The present work examines the relationship between objectively measured time spent in sedentary behaviour/PA of different intensity and eating behaviour traits in young, healthy adults. It also examines the effect of a six-month exercise training program of different intensity on the eating behaviour traits of the same group of subjects.

## 2. Materials and Methods 

### 2.1. Participants and Design

**Figure 1:**
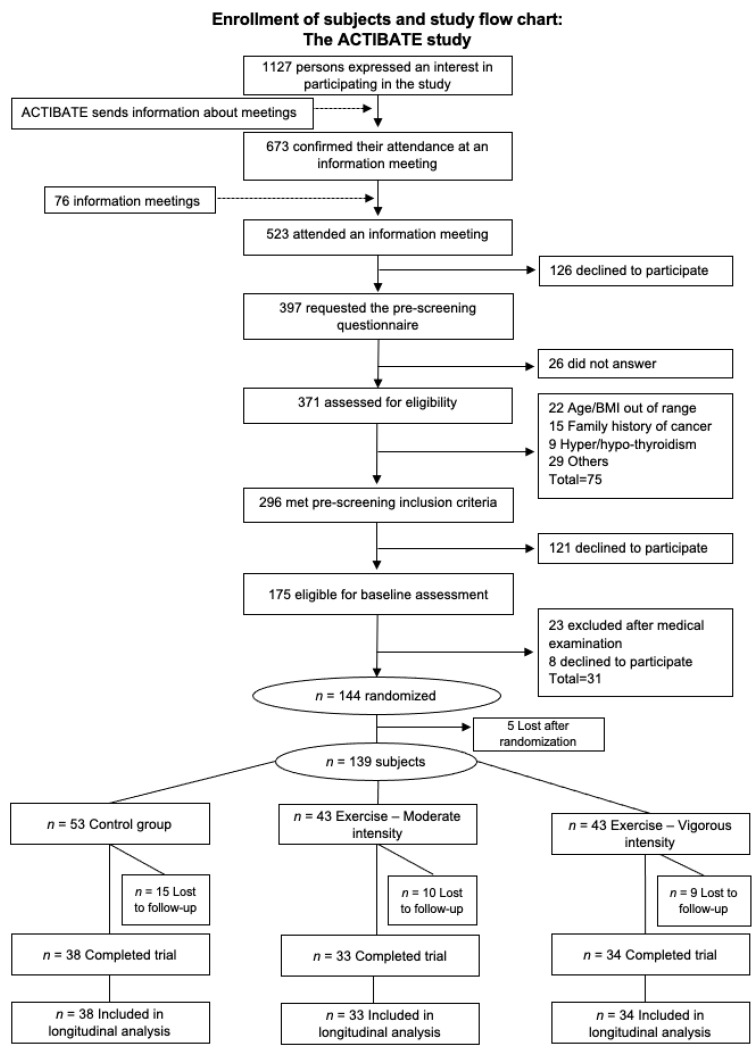
Enrollment of subjects and study flow chart. BMI—body mass index.

A total of 139 young adults (age 18–25 years, 22 ± 2 years; 68.6% women), all non-smokers, were enrolled in the present study (the ACTIBATE study; ClinicalTrials.gov ID: NCT02365129) [15]. Their BMI lay between 18.5 and 35 kg/m^2^. None took any medication, all reported a stable body weight over the preceding 3 months (<3 kg change), and all had a normal electrocardiogram. They reported themselves to be sedentary (<20 min physical activity on <3 days/week). Subjects were recruited via social networks and local media. Interested parties came to information meetings at which the aims of the main study were explained, together with the measurements to be taken, the requirements of the participants, and the types of intervention. Those who wished to take part provided written informed consent to that effect and underwent a medical examination before entering the study. The study protocol and design were approved by the Ethics Committee on Human Research of the University of Granada (no. 924) and the Andalusian Health Service (SAS), and adhere to the Declaration of Helsinki (2013 revision). Figure 1 shows the study flow-chart.

### 2.2. Assessment of Time Spent in Sedentary Behaviour and in Physical Activity 

To objectively measure the time the subjects spent in sedentary behaviour and in PA, participants were asked to wear a wrist-worn GT3X+ model accelerometer (ActiGraph, Pensacola, FL, USA) for 7 consecutive days (24 h/day). Subjects came to the research centre and were given instructions regarding the wearing of the accelerometer and told to remove it only during activities such as bathing or swimming. The accelerometers were initialized to store raw accelerations at a sampling frequency of 100 Hz. Raw accelerations were exported using ActiLife v6.13.3 software (ActiGraph, Pensacola, FL, USA) and converted to .csv format. The raw .csv files were then imported into R software v3.1.2 (https://www.cran.r-project.org/) and processed using GGIR software v1.5-12 (https://cran.r-project.org/web/packages/GGIR/). The latter involved: 1) auto-calibration of the data according to local gravity [16]; 2) calculation of the Euclidean Norm Minus One (ENMO) as Equation (1) with negative values rounded to zero; 3) Detection of non-wear time based on the raw acceleration of the three axes; briefly each 15-min block was classified as non-wear time if the standard deviation of 2 of the 3 axes was <13 mG during the surrounding 60 min moving-time window, or if the value range for 2 of the 3 axes was <50 mG; 4) Detection of sustained abnormally high accelerations, i.e., >5.5 G, related to malfunctioning of the accelerometers; 5) imputation of detected non-wear time and abnormally high accelerations; 6) identification of waking and sleeping hours via an automated algorithm guided by participants’ diary reports [17]; and 7) estimation of time spent in sedentary and PA behaviour using age-specific cut-points for Euclidean norm minus one (ENMO) [18,19]. Only participants wearing the accelerometers for ≥16 h/day and ≥4 h/night over at least 4 days (including at least 1 weekend day) were included in analyses. Additionally, the time spent in moderate-vigorous PA in bouts of ≥10 min (MVPA10min), with a drop-down tolerance of 2 min, was calculated. These categories were established according to the World Health Organization PA recommendations for adults [20]. The mean daily ENMO (mG) (only waking time) was used as a general indicator of PA level.

(√(x^2^ + y^2^ + z^2^)) – 1G (where 1G ~ 9.8 m/s^2^) (1)

### 2.3. Eating Behaviour Traits 

Eating behaviour traits were recorded using the following self-answered questionnaires:

- The Binge Eating Scale (BES). This was used to assess eight feelings/cognitive actions (e.g., guilt, worry over excess eating of certain foods) and eight behavioural manifestations (e.g., eating rapidly, eating in secret) related to binge eating. The weight of each statement (0–3) is then summed. A higher total score reflects more severe binge-eating problems [8].
- The Three-Factor Eating Questionnaire-R18 (TFEQ). This was used to assess three “dimensions” of eating behaviour: (i) cognitive restraint (six questions), i.e., the conscious restriction of food intake in order to control body weight or to promote weight loss; (ii) uncontrolled eating (nine questions), i.e., the tendency to eat more than usual due to a loss of control over intake, accompanied by subjective feelings of hunger, and (iii) emotional eating (three questions), characterized by the inability to resist emotional cues, or eating as a response to different negative emotions. This questionnaire involves 18 questions each measured on a 4-point response scale (definitely true: 1, mostly true: 2, mostly false: 3, definitely false: 4). The scores are summed for each dimension. This shortened version of the original questionnaire maintains its validity and internal consistency [10].
- Control of Eating Questionnaire (CoEQ). This questionnaire comprises 21 questions designed to assess the type and intensity of food cravings experienced, as well as subjective sensations regarding appetite and mood [11]. Subjects answered according to their experiences over the previous seven days; all answers were provided using a visual analogue scale (0–100). The scores for the subscales Craving Control, Craving for Sweet, Craving for Savoury and Positive Mood were then calculated.

### 2.4. Anthropometric and Body Composition Assessments 

Body weight and height were measured using a SECA model 799 scale and stadiometer (SECA, Hamburg, Germany). Both measurements were taken with subjects barefoot and wearing light clothes. BMI was calculated as body mass divided by height^2^ (kg/m^2^). Body composition was measured using a Discovery Wi dual-energy X-ray absorptiometer (DXA) (Hologic, Inc., Bedford, MA, USA). The fat mass index (FMI) and lean mass index (LMI) were calculated as fat/lean mass divided by height^2^ (kg/m^2^).

### 2.5. Energy Intake 

Habitual food intake was recorded via three non-consecutive 24 h recalls (two weekdays and one weekend day/holiday) during face-to-face interviews with a trained dietician. The interviews were meal-sequence-based and involved a detailed assessment and description of all foods consumed. During the interviews, photographs of different portion sizes were used to improve the accuracy of food quantification [21]. Energy and nutrient intakes were then determined using EvalFINUT^®^ software, which is based on the United States Department of Agriculture (USDA) and Spanish Food Composition (BEDCA) databases.

### 2.6. Exercise Intervention

The subjects were assigned (by simple randomization with the researchers blinded to the process) to either (1) a control group (usual lifestyle); (2) a moderate-intensity exercise intervention group in which subjects undertook 150 min/week of aerobic exercise at 60% of the heart rate reserve (HRres), plus two sessions per week of resistance training at 50% of the 1 repetition maximum (RM), for six months; (3) a vigorous-intensity exercise intervention group, in which subjects performed 75 min/week aerobic exercise at moderate intensity (60% HRres) and 75 min/week at a vigorous intensity (80% HRres) plus two sessions per week of resistance training at 70% of the 1 repetition maximum (RM), for six months [15]. 

All subjects were asked to attend 3–4 training sessions per week. Resistance training was performed only in two sessions, while aerobic exercise was performed in every session. Aerobic exercise included the use of a cycle ergometer, treadmill and elliptical ergometer. The strength-training programme was mainly focused on the major upper and lower body muscle groups [15]. To achieve the required volume and intensity of activity, the training programme involved gradual progression towards the assigned exercise intensities, starting with a familiarization period followed by four incremental phases until the assigned intensities were reached [15]. Subjects were asked to wear a heart rate monitor (RS800CX, Polar Electro Öy, Kempele, Finland) during all training sessions to ensure compliance. Graduates in sport sciences directed all the training sessions at the Sport and Health University Research Institute (iMUDS), Granada, Spain (max. 16 subjects per session), except when—if necessary—subjects were allowed to perform their training exercise sessions at home. In such instances, the heart rate monitors were used to assess compliance to the aerobic training and resistance training was adapted to elastic band and weight-bearing exercises and its compliance self-reported. All subjects were asked to maintain their habitual diet and not to undertake additional sporting activities. Subjects could withdraw at any time. At the end of the intervention period, the subjects completed the above-mentioned questionnaires once more. Detailed information about the exercise programme can be found elsewhere [15].

### 2.7. Statistical Analysis 

Descriptive data are presented as means ± standard deviations and were subject to analyses of normality, skewness and kurtosis. Bivariate correlations and simple linear regression (model 0) were used to study the association between eating behaviour traits and objectively measured time spent in sedentary behaviour/PA of different intensity. Partial correlations were also performed adjusting for sex. Multiple linear regression adjusted for sex (model 1), for sex and habitual energy intake (model 2), for sex and BMI (model 3), and for sex and lean mass (model 4) were also performed.

Two-factor (stage (i.e., pre/post intervention) and group) mixed analysis of variance (ANOVA) was used to study changes in eating behaviour traits after the exercise intervention. Additionally, a one-factor analysis of covariance (ANCOVA) was performed to compare the change (post/pre) in eating behaviour traits (dependent variable) between groups (fixed factor), adjusting for the corresponding baseline values. Bonferroni post hoc tests with adjustment for multiple comparisons were used to study the differences in post/pre-intervention values between intervention groups. Significance was set at *p* < 0.05. All calculations were performed using the Statistical Package for Social Sciences (SPSS, v. 22.0, IBM SPSS Statistics, IBM Corporation). The GraphPad Prism 5 package (GraphPad Software, San Diego, CA, USA) was used to construct plots.

## 3. Results

Table 1 shows the characteristics of participants at baseline, including eating behaviour traits as determined by BES, TFEQ and CoEQ.

### 3.1. Cross-Sectional Analyses

Table 2 shows the associations between the time spent in sedentary behaviour/habitual PA of different intensity and eating behaviour traits.

**Figure 2:**
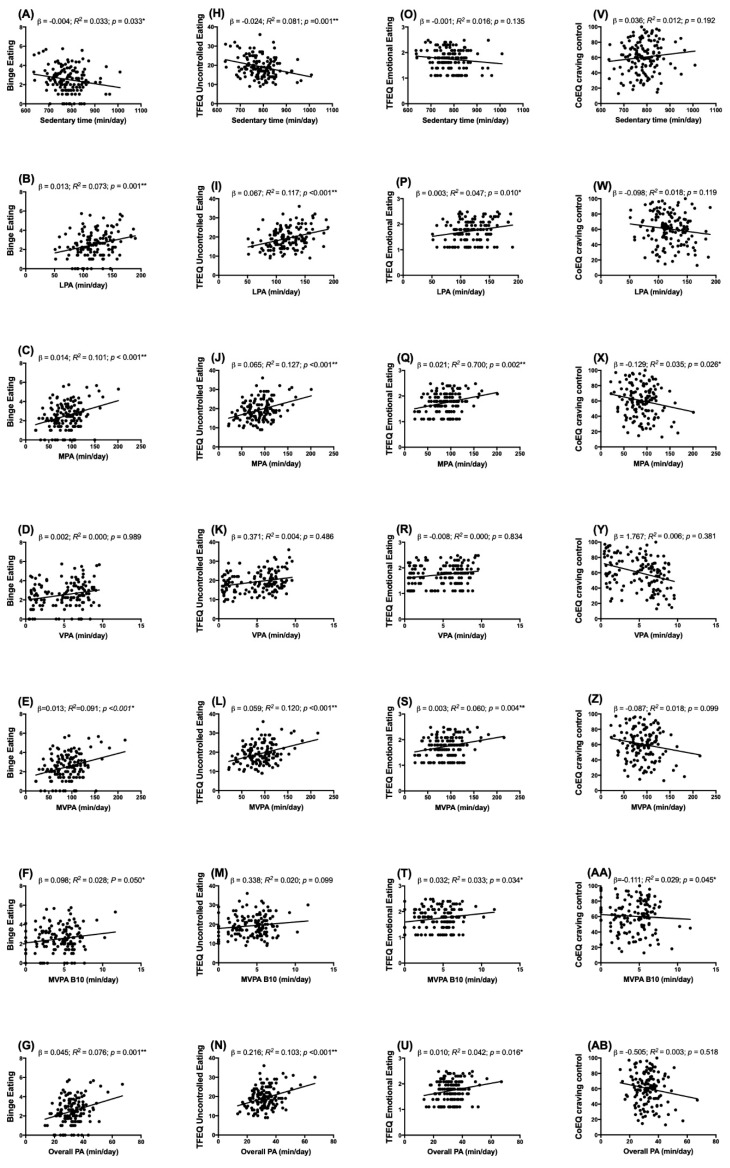
Association between Binge Eating (Panel **A**–**G**)**,** Three-Factor Eating Questionnaire Uncontrolled Eating (Panel (**H**–**N**), and Emotional Eating (Panel (**O**–**U**)**,** and Control of Eating Questionnaire Craving Control (Panel **V**–**AB**) and time spent in sedentary behaviour and Physical Activity of different intensity. Unstandardized simple regression coefficient (*β*) and standardized coefficients of determination (*R^2^*) are provided. LPA—Light Physical Activity; MPA—Moderate Physical Activity; VPA—Vigorous Physical Activity; MVPA—Moderate-to-vigorous Physical Activity; MVPA B10—Moderate-to-vigorous Physical Activity in bouts of ten minutes. (Model 0, *n* = 139). * *p* < 0.05, ** *p* < 0.01.

Sedentary behaviour was inversely associated with binge eating (*p* < 0.05) and uncontrolled eating (*p* = 0.001). Light PA (*p* = 0.001), moderate PA (*p* < 0.001), moderate-to-vigorous PA (*p* < 0.001), moderate-to-vigorous in bouts of 10 min (*p* < 0.05) and overall PA (*p* = 0.001), were positively associated with binge eating, uncontrolled eating and emotional eating (Table 2). Moreover, Moderate PA (*p* < 0.05) and Moderate-to-vigorous PA (*p* < 0.05) were inversely associated with craving control. No associations were found with other dimensions of eating behaviour traits (i.e., cognitive restraint, craving for sweet, craving for savoury and positive mood). VPA was associated with none of the eating behaviour-trait dimensions. Figure 2 shows the dispersion values for those eating behaviour traits variables that were significantly correlated with time spent in sedentary behaviour/PA of different intensity. All associations remained significant after adjusting for sex (model 1), sex and energy intake (model 2), sex and BMI (model 3), and sex and lean mass (model 4) (Supplementary Table S1).

Supplementary Table S2 shows the relationships between eating behaviour traits and body composition. Binge eating was correlated with BMI, lean mass, fat mass and visceral adipose tissue mass (all *p* < 0.05). Cognitive restraint was only associated with fat mass. Supplementary Table S3 shows the associations between eating behaviour traits and PA intensity by sex. In women, binge eating, and uncontrolled eating were associated with sedentary behaviour and PA intensity. Uncontrolled eating and emotional eating were associated with sedentary behaviour and PA intensity in men (all *p* < 0.05).

### 3.2. Longitudinal Analysis 

A total of 105 participants were included in the longitudinal analyses (loss to follow-up = 24.5%) (Figure 1). It includes 105 subjects who attended at least 70% of the training sessions. 

**Figure 3:**
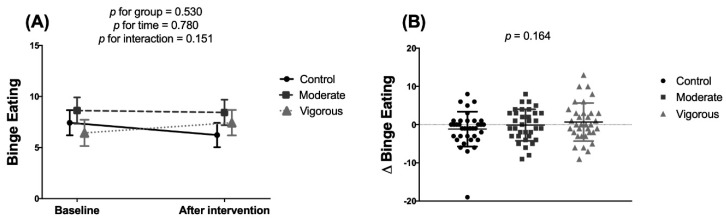
Effects of exercise intervention on Binge Eating in young adults. Panel (**A**) shows the results of two-factor mixed analysis of variance (ANOVA). Panel (**B**) shows a one-factor analysis of covariance (ANCOVA) comparing post-pre differences (adjusted for the baseline value). Control group *n* = 35; Moderate-intensity group *n* = 32; Vigorous-intensity group *n* = 32. Values are adjusted means and standard error.

**Figure 4:**
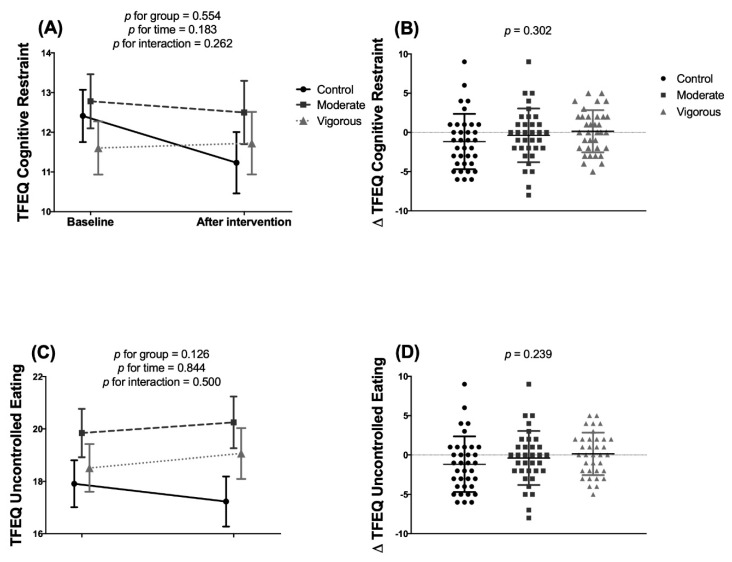

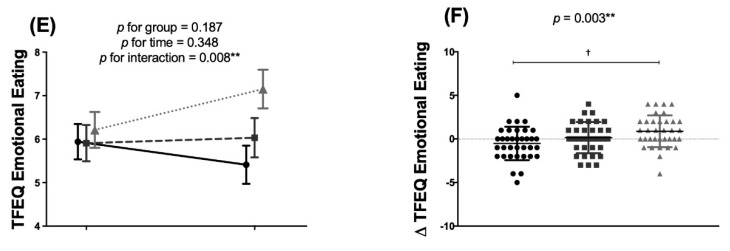
Effects of exercise intervention on Three-Factor Eating Questionnaire variables in young adults. Panels (**A**,**C**,**E**): two-factor mixed ANOVA. Panels (**B**,**D**,**F**): a one-factor one-factor analysis of covariance (ANCOVA) comparing post-pre differences, adjusted for baseline value. Control group *n* = 34; Moderate intensity group *n* = 32; Vigorous-intensity group *n* = 33. Values are adjusted means and standard error. † Simbol indicates significant differences (post-hoc comparisons). ** *p* < 0.01

The intervention treatments had no significant effect on binge eating (Figure 3), cognitive restraint (Figure 4A,B) or uncontrolled eating (Figure 4C,D) but did have a significant effect on emotional eating (Figure 4E,F) (*p* = 0.008 two-factor mixed ANOVA, and *p* = 0.003 ANCOVA). Post-hoc analyses revealed significant differences between the control and vigorous-intensity groups with respect to emotional eating, which was increased in the latter group (mean difference 1.53, 95% confidence interval [CI] 0.452–2.619, *p* = 0.002).

**Figure 5:**
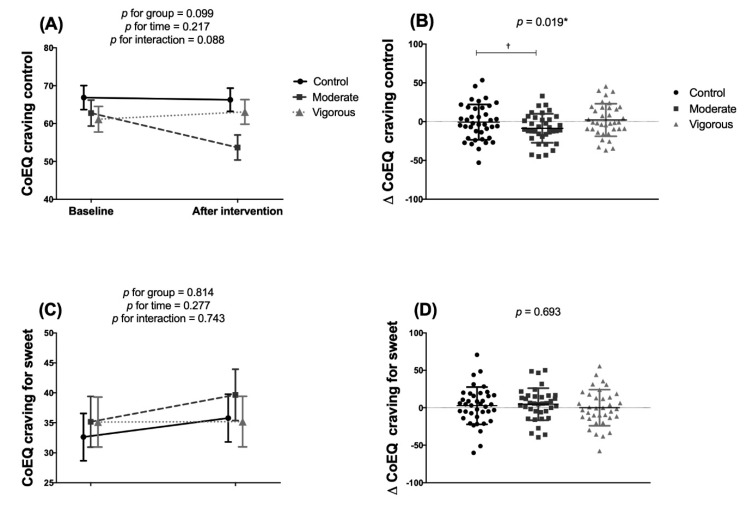

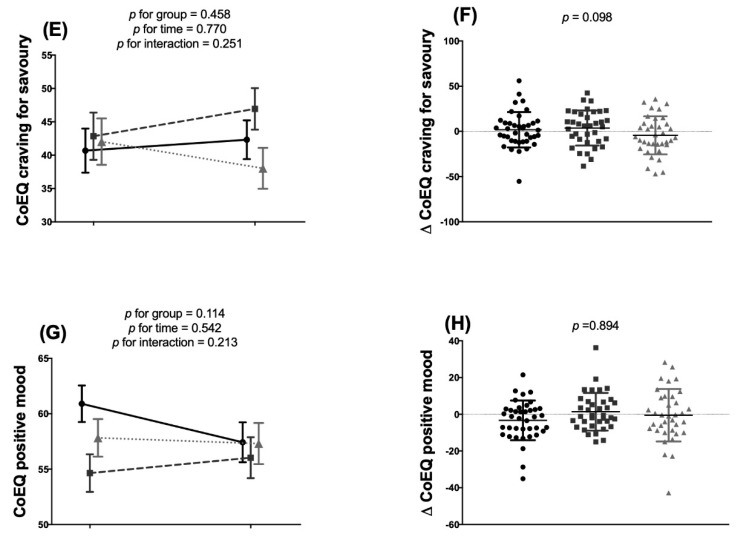
Effects of exercise intervention on Control of Eating Questionnaire variables in young adults. Panels (**A**,**C**,**E**,**G**) two-factor mixed ANOVA. Panels (**B**,**D**,**F**,**H**): one-factor ANCOVA comparing post-pre differences, adjusted for baseline value. Control group *n* = 38; Moderate intensity group *n* = 33; Vigorous-intensity group *n* = 34. Values are adjusted means and standard error. ^†^ Simbol indicates significant differences (post-hoc comparisons).* *p* < 0.05.

No significant effect of either intervention treatment was seen on the CoEQ subscales as examined by two-factor mixed ANOVA (Figure 5A,C,E,G). ANCOVA detected no effect on craving for sweet (Figure 5C,D), craving for savoury (Figure 5E,F), or positive mood (Figure 5G,H), but revealed significant differences between the control and moderate exercise groups in terms of craving control (mean difference 11.03, 95%CI 0.846–21.205, *p* = 0.019) (Figure 5B). 

## 4. Discussion

The present results show that binge eating, uncontrolled eating and emotional eating are inversely associated with time spent in sedentary behaviour, and directly associated with time spent in PA, especially MVPA and overall PA in young, healthy adults. However, these eating behaviour traits were not altered by a six-month exercise training intervention (combining aerobic and resistance training at different intensities), which even significantly increased emotional eating in those following the vigorous-intensity exercise program. The lack of exercise-induced effects on binge and uncontrolled eating suggests that PA did not influence these psychological markers, maybe due to their lasting nature.

### 4.1. Association of Eating Behaviour Traits with Time Spent in Sedentary Behaviour and Physical Activity 

In the present work, sedentary behaviour was inversely associated with questionnaire reports of binge eating and uncontrolled eating. This study also shows that young healthy adults who are more physically active, are more likely to consciously self-report a tendency to binge eat and eat uncontrollably—perhaps because they understand PA as deserving of reward [22]. According to a study done by Sim et al. [23], inactive overweight restrained-eaters appear to adjust their eating behaviour depending on the perceived healthiness of activities like exercising. It follows the Compensatory Health Beliefs model by Rabiau et al. which states that the negative effects of an unhealthy behaviour can be compensated for another healthy behaviour [24]. Our findings disagree however with those by Shook et al. [25] who reported low-level PA to be associated with higher levels of appetite-related disinhibition, and that less active individuals show significantly more intense cravings for savoury foods (e.g., chips, burgers and pizza). The relationship between physical activity and appetite control may vary from culture to culture and between geographical regions and needs further research. The present results show body composition to be associated with binge eating. This is similar to that reported by Myers et al. [26], who concluded that a relationship exists between adiposity and uncontrolled and binge eating. 

### 4.2. Effect of the Exercise Intervention on Eating Behaviour Traits 

The only eating behaviour trait modified by the exercise intervention was emotional eating (assessed by the self-reported TFEQ), which increased in the vigorous-intensity exercise group. It suggests that exercise might have negatively affected the ability to resist emotional cues or eating as a response to different negative emotions. This might be explained by the effect of training on emotional state and mood. Hormonal changes that occur during exercise can affect mood positively and reduce stress indicators [27]. Although the response may differ between individuals. Emotional eating has been associated with individuals with lower self-esteem and poorer perception of their physical fitness [10]. Emotional eating can manifest as either increased food intake or food avoidance [28]. In the present work, the subjects of the moderate-intensity exercise group showed a trend towards reduced craving control, suggesting overeating may be more likely than food avoidance [11]. 

We did not find any other effect of this intervention in this population. This suggests that the numerous associations between eating behaviour traits and physical activity levels observed at baseline are not reflecting a cause–effect relationship and are likely spurious or explained by other confounders. It is also relevant to consider that the eating behaviour traits represent more lasting and resilient influences on the tendency to eat or on food selection, and are not modified on a daily basis, and they influence food consumption in different ways and through different processes [29].

The present work suffers from the possible limitation that the paper-based CoEQ was completed during the baseline and post-intervention measurement visit in the presence of a nutritionist whom the subjects may have tried to please, introducing a certain bias into their answers. In addition, all subjects saw all three questionnaires twice, once at baseline and again after the intervention. This may have introduced some ‘learning’ bias. Not previously validated Spanish versions of the BES and CoEQ were used.

## 5. Conclusions

The findings of this study have revealed counterintuitive associations between eating behaviour traits of binge eating, uncontrolled eating and emotional eating with sedentary behaviour and physical activity in this population. In contrast, these self-reported eating behaviours were not modified by an exercise program training, which suggests that the aforementioned counterintuitive associations were not reflecting a cause–effect relationship. Further studies are needed to better understand these findings between the enduring nature of eating traits and physical activity.

## Figures and Tables

**Table 1:** Characteristics of participants at baseline.

		All (*n* = 139)	CG (*n* = 53)	MIIG (*n* = 43)	VIIG (*n* = 43)
**Women (*n*, (%))**		95, (68.35)	34, (64.20)	31, (72.10)	30, (69.80)
Men (*n*, (%))		44, (31.65)	19, (35.80)	12, (27.90)	13, (30.20)
Age (years)		22.06 ± 2.26	21.8 ± 2.17	22.08 ± 2.19	22.35 ± 2.45
**Body composition**					
BMI (kg/m^2^) ^a^		24.95 ± 4.57	24.47 ± 5.03	25.58 ± 4.13	24.91 ± 4.40
Lean mass (kg) ^b^		41.22 ± 9.15	41.43 ± 10.11	40.90 ± 8.05	41.28 ± 9.12
Fat mass (kg) ^b^		25.03 ± 8.65	23.87 ± 8.75	26.90 ± 8.77	24.65 ± 8.29
Fat mass (%) ^b^		36.08 ± 7.45	34.95 ± 7.34	37.95 ± 8.04	35.65 ± 6.76
**Eating behaviour traits**					
Binge Eating (BES)		8.10 ± 7.25	7.81 ± 7.87	9.4 ± 6.27	7.16 ± 7.35
TFEQ	Cognitive Restraint	12.07 ± 3.78	12.34 ± 3.39	12.56 ± 4.15	11.26 ± 3.82
	Uncontrolled Eating	19.32 ± 5.43	19.09 ± 5.49	19.79 ± 4.89	19.14 ± 5.95
	Emotional Eating	6.17 ± 2.43	6.08 ± 2.48	6.16 ± 6.16	6.28 ± 2.36
CoEQ	Craving control	60.34 ± 20.60	62.34 ± 20.82	57.83 ± 19.50	60.40 ± 21.56
	Craving for sweet	36.65 ± 24.23	36.64 ± 25.03	36.61 ± 23.11	36.71 ± 24.90
	Craving for savoury	44.47 ± 19.96	44.35 ± 22.89	44.85 ± 17.18	44.23 ± 19.12
	Positive mood	58.15 ± 11.72	61.07 ± 12.43	54.89 ± 10.81	57.80 ± 11.02
**Time spent in sedentary behaviour/habitual PA intensity**					
Valid days (days)		6.77 ± 0.54	6.77 ± 0.54	6.74 ± 0.58	6.79 ± 0.51
Wear time (min/day)		19.91 ± 25.59	19.30 ± 24.02	18.80 ± 29.37	21.80 ± 23.85
Waking time (min/day)		995.86 ± 49.56	992.47 ± 57.30	1009.66 ± 45.45	986.22 ± 40.42
Sedentary time (min/day)		785.40 ± 63.88	788.38 ± 71.10	788.71 ± 63.50	778.43 ± 55.18
LPA (min/day)		119.40 ± 27.85	114.90 ± 28.08	124.72 ± 27.48	119.64 ± 27.63
MPA (min/day)		88.19 ± 30.02	86.63 ± 33.59	92.87 ± 26.91	85.43 ± 28.42
VPA (min/day)		2.86 ± 3.50	2.57 ± 3.28	3.36 ± 4.25	2.71 ± 2.83
MVPA (min/day)		91.05 ± 31.61	89.19 ± 35.24	96.23 ± 28.37	88.15 ± 30.02
MVPA B_10_ (min/day)		24.00 ± 20.64	26.20 ± 22.10	23.49 ± 15.34	21.80 ± 23.44
Overall PA (ENMO, mG/5s)		32.56 ± 8.06	31.72 ± 8.76	33.68 ± 7.45	32.49 ± 7.81

Data are presented as means and standard deviation. ^a^ *n* = 135; ^b^ *n* = 124. CG—control group; MIIG—moderate-intensity intervention group; VIIG—vigorous-intensity intervention group; BMI—body mass index; BES—binge eating scale; TFEQ—Three-Factor Eating Questionnaire; CoEQ—Control of Eating Questionnaire; PA—physical activity; LPA—light PA; MPA—moderate PA; VPA—vigorous PA; MVPA—moderate-to-vigorous PA; MVPA B_10_—moderate-to-vigorous PA in bouts of ten minutes; ENMO—Euclidean norm minus one. All baseline data are similar across study groups (all *p* > 0.05).

**Table 2:** Bivariate correlations between eating behaviour traits and time spent in sedentary behaviour and habitual physical activity (PA) intensities.

	Binge Eating	TFEQ			CoEQ			
		CR	UE	EE	Craving Control	Craving for Sweet	Craving for Savoury	Positive Mood
**Sedentary Time**	−0.181 *	−0.031	−0.286 **	−0.127	0.111	−0.073	−0.025	−0.071
**LPA**	0.270 **	0.107	0.340 ***	0.218 *	−0.133	0.090	0.088	0.028
**MPA**	0.317 ***	0.095	0.359 ***	0.264 **	−0.188 *	0.115	0.121	−0.071
**VPA**	0.001	−0.001	0.060	−0.018	0.075	−0.061	−0.050	0.091
**MVPA**	0.302 ***	0.091	0.346 ***	0.245 **	−0.170 *	0.105	0.109	−0.059
**MVPA B_10_**	0.167 *	0.152	0.140	0.180 *	−0.055	0.124	−0.014	−0.092
**Overall PA (ENMO, mG/5s)**	0.275 **	0.059	0.321 ***	0.204 *	−0.152	0.085	0.071	−0.015

Values are Pearson correlation coefficients (*r*); * *p* < 0.05; ** *p* < 0.01; *** *p* < 0.001; TFEQ—Three-Factor Eating Questionnaire; CR—cognitive restraint; UE—uncontrolled eating; EE—emotional eating; CoEQ—Control of Eating Questionnaire; LPA—Light Physical Activity; MPA—Moderate Physical Activity; VPA—vigorous Physical Activity; MVPA—moderate-to-vigorous Physical Activity; MVPA B_10_—moderate-to-vigorous Physical Activity in bouts of ten minutes; ENMO—Euclidean norm minus one.

## Supplementary Materials

The following are available online at https://www.mdpi.com/2072-6643/12/12/3685/s1, Table S1: Relationships between times spent in sedentary behaviour/habitual PA at different intensity and eating behaviour traits., Table S2. Bivariate correlations between eating behaviour traits and body composition in young adults., Table S3. Bivariate correlations between eating behaviour traits and time spent in sedentary behaviour/physical activity of different intensity in young men and women.
