# Supplementary material for: Eating Behavior, Physical Activity and Exercise Training: A Randomized Controlled Trial in Young Healthy Adults
Source: Nutrients. 2020 Nov 29;12(12):3685. doi: 10.3390/nu12123685 (PMC7760390; doi:10.3390/nu12123685)
Supplement: Supplementary file 1 [file nutrients-12-03685-s001.pdf]

SUPPLEMENTARY MATERIAL

Supplementary Table 1. Relationships between times spent in sedentary behaviour/habitual PA at different intensity and eating behaviour traits.

Binge eating scale

	Model 0			Model 1			Model 2			Model 3			Model 4		
	β	R^2	P	β	R^2	P	β	R^2	P	β	R^2	P	β	R^2	P
Sedentary time (min/day)	-0.004	0.033	**0.033**	-0.004	0.033	**0.035**	-0.006	0.123	**0.012**	-0.005	0.118	**0.006**	-0.004	0.117	**0.026**
LPA (min/day)	0.013	0.073	**0.001**	0.013	0.075	**0.001**	0.015	0.134	**0.008**	0.013	0.138	**0.001**	0.012	0.131	**0.009**
MPA (min/day)	0.014	0.101	**0.000**	0.014	0.101	**0.000**	0.018	0.239	**0.000**	0.014	0.170	**0.000**	0.012	0.147	**0.003**
VPA (min/day)	0.002	0.000	0.989	0.000	0.000	0.999	0.023	0.024	0.885	0.030	0.065	0.815	0.003	0.080	0.981
MVPA (min/day)	0.013	0.091	**0.000**	0.013	0.091	**0.000**	0.016	0.215	**0.000**	0.013	0.161	**0.000**	0.011	0.142	**0.004**
MVPA B$_{10}$ (min/day)	0.098	0.028	0.050	0.097	0.028	0.052	0.133	0.079	0.063	0.105	0.096	**0.033**	0.060	0.090	0.246
Overall PA (ENMO, mG/5s)	0.045	0.076	**0.001**	0.046	0.076	**0.001**	0.052	0.157	**0.003**	0.049	0.152	**0.000**	0.042	0.141	**0.004**

Three Factor Eating Questionnaire - Cognitive Restraint

	Model 0			Model 1			Model 2			Model 3			Model 4		
	β	R^2	P	β	R^2	P	β	R^2	P	β	R^2	P	β	R^2	P
Sedentary time (min/day)	-0.002	0.001	0.715	0.000	0.029	0.956	0.004	0.048	0.634	-0.002	0.042	0.686	0.000	0.029	0.965
LPA (min/day)	0.015	0.012	0.208	0.010	0.034	0.399	0.002	0.045	0.909	0.009	0.045	0.428	0.011	0.034	0.420
MPA (min/day)	0.012	0.009	0.268	0.010	0.034	0.375	0.004	0.046	0.783	0.009	0.046	0.405	0.005	0.030	0.657
VPA (min/day)	-0.005	0.000	0.989	-0.049	0.029	0.895	0.117	0.046	0.806	0.009	0.046	0.405	0.016	0.029	0.969
MVPA (min/day)	0.011	0.008	0.289	0.009	0.009	0.404	0.005	0.047	0.747	0.008	0.045	0.425	0.005	0.030	0.649
MVPA B$_{10}$ (min/day)	0.255	0.023	0.075	0.239	0.049	0.092	0.280	0.071	0.197	0.237	0.061	0.096	0.159	0.037	0.303
Overall PA (ENMO, mG/5s)	0.028	0.003	0.492	0.016	0.030	0.689	0.004	0.045	0.938	0.019	0.042	0.641	0.010	0.029	0.819

Three Factor Eating Questionnaire - Uncontrolled Eating

	Model 0			Model 1			Model 2			Model 3			Model 4		
	β	R^2	P	β	R^2	P	β	R^2	P	β	R^2	P	β	R^2	P
Sedentary time (min/day)	-0.024	0.081	**0.001**	-0.025	0.085	**0.001**	-0.033	0.172	**0.001**	-0.026	0.093	**0.000**	-0.025	0.103	**0.001**
LPA (min/day)	0.067	0.117	**0.000**	0.071	0.126	**0.000**	0.075	0.183	**0.001**	0.076	0.143	**0.000**	0.068	0.122	**0.000**
MPA (min/day)	0.065	0.127	**0.000**	0.066	0.133	**0.000**	0.075	0.218	**0.000**	0.070	0.146	**0.000**	0.060	0.123	**0.000**
VPA (min/day)	0.371	0.004	0.486	0.379	0.004	0.478	0.970	0.052	0.144	0.416	0.008	0.448	0.200	0.017	0.735
MVPA (min/day)	0.059	0.120	**0.000**	0.061	0.123	**0.000**	0.071	0.217	**0.000**	0.064	0.136	**0.000**	0.055	0.114	**0.000**
MVPA B$_{10}$ (min/day)	0.338	0.020	0.099	0.342	0.020	0.097	0.515	0.064	0.090	0.343	0.023	0.106	0.218	0.024	0.323
Overall PA (ENMO, mG/5s)	0.216	0.103	**0.000**	0.223	0.108	**0.000**	0.270	0.215	**0.000**	0.233	0.118	**0.000**	0.207	0.104	**0.001**

Three Factor Eating Questionnaire - Emotional Eating															
	Model 0			Model 1			Model 2			Model 3			Model 4		
	β	R^2	P	β	R^2	P	β	R^2	P	β	R^2	P	β	R^2	P
Sedentary time (min/day)	-0.001	0.016	0.135	-0.001	0.031	0.208	-0.001	0.036	0.197	-0.026	0.093	**0.000**	-0.001	0.054	0.108
LPA (min/day)	0.003	0.047	**0.010**	0.003	0.056	**0.023**	0.005	0.124	**0.007**	0.076	0.143	**0.000**	0.003	0.072	**0.027**
MPA (min/day)	0.021	0.700	**0.002**	0.003	0.081	**0.003**	0.005	0.134	**0.005**	0.003	0.091	**0.002**	0.003	0.084	**0.011**
VPA (min/day)	-0.008	0.000	0.834	-0.012	0.020	0.758	0.014	0.010	0.782	-0.009	0.025	0.824	0.012	0.034	0.788
MVPA (min/day)	0.003	0.060	**0.004**	0.003	0.072	**0.006**	0.004	0.117	**0.009**	0.003	0.082	**0.005**	0.003	0.080	**0.014**
MVPA B_{10} (min/day)	0.032	0.033	**0.034**	0.031	0.049	**0.041**	0.014	0.014	0.553	0.032	0.056	**0.039**	0.027	0.056	0.088
Overall PA (ENMO, mG/5s)	0.010	0.042	**0.016**	0.009	0.054	**0.028**	0.012	0.077	**0.039**	0.010	0.064	**0.021**	0.011	0.076	**0.019**

Control of Eating Questionnaire - Craving Control															
	Model 0			Model 1			Model 2			Model 3			Model 4		
	β	R^2	P	β	R^2	P	β	R^2	P	β	R^2	P	β	R^2	P
Sedentary time (min/day)	0.036	0.012	0.192	0.030	0.025	0.276	0.057	0.064	0.195	0.038	0.045	0.182	0.033	0.044	0.266
LPA (min/day)	-0.098	0.018	0.119	-0.081	0.028	0.207	-0.111	0.059	0.212	-0.075	0.042	0.248	-0.070	0.042	0.332
MPA (min/day)	-0.129	0.035	**0.026**	-0.120	0.046	**0.039**	-0.143	0.080	0.106	-0.126	0.066	**0.032**	-0.120	0.063	0.058
VPA (min/day)	1.767	0.006	0.381	1.950	0.023	0.332	2.801	0.055	0.310	2.175	0.041	0.281	1.657	0.038	0.460
MVPA (min/day)	-0.087	0.018	0.099	-0.102	0.040	0.066	-0.116	0.069	0.165	-0.107	0.059	0.055	-0.105	0.058	0.081
MVPA B_{10} (min/day)	-0.111	0.029	**0.045**	-0.438	0.018	0.574	-0.225	0.039	0.860	-0.622	0.037	0.428	-0.586	0.038	0.487
Overall PA (ENMO, mG/5s)	-0.505	0.003	0.518	-0.348	0.034	0.112	-0.301	0.053	0.350	-0.354	0.051	0.108	-0.384	0.054	0.110

Control of Eating Questionnaire - Craving for Sweet															
	Model 0			Model 1			Model 2			Model 3			Model 4		
	β	R^2	P	β	R^2	P	β	R^2	P	β	R^2	P	β	R^2	P
Sedentary time (min/day)	-0.003	0.005	0.390	-0.001	0.073	0.705	-0.003	0.135	0.465	-0.001	0.073	0.703	-0.001	0.096	0.732
LPA (min/day)	0.007	0.008	0.293	0.002	0.073	0.716	0.001	0.127	0.955	0.003	0.073	0.695	0.002	0.095	0.770
MPA (min/day)	0.009	0.013	0.177	0.006	0.079	0.312	0.009	0.141	0.329	0.007	0.080	0.291	0.005	0.100	0.423
VPA (min/day)	-0.154	0.004	0.478	-0.195	0.078	0.353	-0.209	0.135	0.456	-0.222	0.079	0.300	-0.242	0.103	0.305
MVPA (min/day)	0.007	0.011	0.219	0.005	0.078	0.378	0.007	0.138	0.392	0.005	0.077	0.363	0.004	0.098	0.480
MVPA B_{10} (min/day)	0.122	0.015	0.146	0.107	0.084	0.190	0.196	0.161	0.126	0.114	0.085	0.171	0.103	0.105	0.246
Overall PA (ENMO, mG/5s)	0.023	0.007	0.322	0.013	0.074	0.587	0.012	0.129	0.711	0.012	0.073	0.609	0.011	0.096	0.668

Control of Eating Questionnaire - Craving for Savoury															
	Model 0			Model 1			Model 2			Model 3			Model 4		
	β	R^2	P	β	R^2	P	β	R^2	P	β	R^2	P	β	R^2	P

Sedentary time (min/day)	-0.001	0.001	0.771	-0.001	0.016	0.602	-0.004	0.026	0.323	-0.001	0.014	0.665	0.000	0.041	0.853
LPA (min/day)	0.005	0.008	0.302	0.007	0.028	0.164	0.005	0.017	0.495	0.006	0.022	0.261	0.004	0.046	0.440
MPA (min/day)	0.007	0.015	0.155	0.007	0.033	0.108	0.008	0.030	0.261	0.007	0.029	0.141	0.005	0.050	0.280
VPA (min/day)	-0.095	0.003	0.555	-0.082	0.016	0.610	-0.149	0.017	0.509	-0.104	0.016	0.524	-0.063	0.042	0.721
MVPA (min/day)	0.006	0.012	0.202	0.006	0.030	0.143	0.007	0.025	0.332	0.006	0.026	0.184	0.005	0.050	0.304
MVPA B_{10} (min/day)	-0.010	0.000	0.866	-0.005	0.014	0.931	-0.041	0.012	0.693	0.001	0.013	0.983	-0.009	0.041	0.891
Overall PA (ENMO, mG/5s)	0.014	0.005	0.408	0.018	0.022	0.292	0.012	0.013	0.639	0.016	0.019	0.367	0.015	0.046	0.442

Control of Eating Questionnaire - Positive Mood

	Model 0			Model 1			Model 2			Model 3			Model 4		
	β	R^2	P	β	R^2	P	β	R^2	P	β	R^2	P	β	R^2	P
Sedentary time (min/day)	-0.013	0.005	0.404	-0.016	0.030	0.261	-0.007	0.009	0.731	-0.013	0.017	0.427	-0.10	0.028	0.542
LPA (min/day)	0.012	0.001	0.740	0.027	0.025	0.461	-0.004	0.007	0.940	0.013	0.013	0.729	0.018	0.027	0.640
MPA (min/day)	-0.028	0.005	0.409	-0.021	0.02	0.528	-0.010	0.008	0.817	-0.028	0.017	0.401	-0.040	0.036	0.239
VPA (min/day)	1.216	0.008	0.289	1.335	0.031	0.242	0.844	0.014	0.528	1.261	0.021	0.267	0.257	0.025	0.831
MVPA (min/day)	-0.022	0.003	0.491	-0.016	0.023	0.624	-0.005	0.007	0.904	-0.022	0.015	0.494	-0.036	0.035	0.261
MVPA B_{10} (min/day)	-0.476	0.008	0.284	-0.433	0.028	0.327	0.184	0.008	0.764	-0.326	0.016	0.461	-0.795	0.050	0.077
Overall PA (ENMO, mG/5s)	-0.022	0.000	0.857	0.009	0.021	0.940	-0.015	0.007	0.926	-0.015	0.012	0.907	-0.088	0.029	0.499

Linear regression analyses were performed without adjusting for any covariate (model 0), and adjusting for sex (model 1), for sex and energy intake (model 2), for sex and BMI (model 3), and for sex and lean mass (model 4). The unstandardized β coefficient, R^2 and P values are provided. PA physical activity, LPA light-PA, MPA moderate-PA, VPA vigorous-PA, MVPA moderate-to-vigorous-PA, MVPA B_{10} moderate-to-vigorous PA in bouts of ten minutes, ENMO Euclidean norm minus one.

Supplementary Table 2. Bivariate correlations between eating behaviour traits and body composition in young adults.

		TFEQ			CoEQ			
	Binge Eating	Cognitive restraint	Uncontrolled eating	Emotional eating	Craving control	Craving for sweet	Craving for savoury	Positive Mood
BMI (kg/m²)	0.259**	0.097	-0.052	0.084	-0.069	-0.098	0.085	0.072
Lean mass (kg)	0.186*	0.001	0.142	0.061	-0.010	-0.105	0.170	0.137
Fat mass (kg)	0.290***	0.174*	-0.073	0.107	-0.071	-0.023	0.046	0.006
Fat mass (%)	0.183*	0.189*	-0.159	0.071	-0.077	0.041	-0.046	-0.008
VAT mass (g)	0.228**	0.128	-0.043	0.057	-0.023	-0.156	0.102	0.110

Values are Pearson correlation coefficients (*r*); * p <0.05); ** p <0.01; *** p <0.001. TFEQ Three-Factor Eating Questionnaire; CoEQ Control of Eating Questionnaire; BMI body mass index; LM lean mass; FM fat mass; VAT visceral adipose tissue.

	Binge eating		TFEQ Cognitive restraint		TFEQ Uncontrolled eating		TFEQ Emotional eating	
	MEN	WOMEN	MEN	WOMEN	MEN	WOMEN	MEN	WOMEN
n	44	95	44	95	44	95	44	95
Sedentary time	-0.140	-0.210*	-0.138	0.074	-0.322*	-0.274**	-0.128	-0.097
LPA	0.262	0.280**	0.190	0.016	0.403**	0.325**	0.285	0.143
MPA	0.351*	0.303**	0.228	0.003	0.347*	0.375***	0.399**	0.170
VPA	0.096	-0.036	-0.064	0.008	0.181	0.008	0.098	-0.079
MVPA	0.334*	0.288**	0.208	0.007	0.346*	0.354***	0.379*	0.153
MVPA B_{10}	0.206	0.148	0.285	0.084	0.067	0.181	0.230	0.146
Overall PA (ENMO, mG/5s)	0.247	0.291**	0.169	-0.028	0.329*	0.328**	0.268	0.144

	CoEQ Craving control		CoEQ Craving for sweet		CoEQ Craving for savoury		CoEQ Positive Mood	
	MEN	WOMEN	MEN	WOMEN	MEN	WOMEN	MEN	WOMEN
n	44	95	44	95	44	95	44	95
Sedentary time	0.018	0.143	-0.082	-0.007	0.016	-0.087	-0.072	-0.117
LPA	-0.106	-0.11	0.179	-0.035	0.198	0.075	0.086	0.048
MPA	-0.133	-0.199	0.146	0.061	0.124	0.146	-0.118	-0.01
VPA	0.010	0.112	-0.063	-0.086	0.029	-0.075	0.178	0.063
MVPA	-0.117	-0.179	0.129	0.053	0.110	0.134	-0.100	-0.003
MVPA B_{10}	0.086	-0.111	-0.014	0.164	-0.119	0.049	-0.219	-0.002

Overall PA (ENMO, mG/5s)	-0.082	-0.163	0.108	0.020	0.072	0.100	-0.007	0.015

Supplementary Table 3. Bivariate correlations between eating behaviour traits and time spent in sedentary behaviour and habitual physical activity of different intensity in young men and women. Values are Pearson correlation coefficients (*r*); *p <0.05); ** p <0.01; ***P<0.001. PA physical activity, LPA light-PA, MPA moderate-PA, VPA vigorous-PA, MVPA moderate-to-vigorous-PA, MVPA B_{10} moderate-to-vigorous-PA in bouts of ten minutes, ENMO Euclidean norm minus one.
